# Clinical and prognostic analysis of 78 patients with human immuno-deficiency virus associated non-Hodgkin’s lymphoma in Chinese population

**DOI:** 10.1186/s13027-017-0120-2

**Published:** 2017-01-23

**Authors:** Yang Shen, Renfang Zhang, Li Liu, Yinzhong Shen, Wei Song, Tangkai Qi, Yang Tang, Zhenyan Wang, Liqian Guan, Hongzhou Lu

**Affiliations:** 1 Department of Hematology, Shanghai Institute of Hematology, Rui Jin Hospital Affiliated to Shanghai Jiao Tong University School of Medicine and Collaborative Innovation Center of Systems Biomedicine, Shanghai Jiao Tong University, Shanghai, 200000 China; 2 Department of Infectious Diseases, Shanghai Public Health Clinical Center, Fudan University, Shanghai, 201508 China

**Keywords:** Human immuno-deficiency virus, Lymphoma, Prognosis, International prognostic index, Prognosis

## Abstract

**Background:**

Human Immuno-deficiency Virus (HIV) associated non-Hodgkin’s lymphoma (NHL) was a special group of disease, which manifests distinct clinical features and prognosis as compared with NHLs in patients without HIV. We performed this study to describe the clinical features of the disease and investigated the potential prognostic factors.

**Methods:**

HIV-infected patients who were newly diagnosed with NHL were enrolled in this study. The selection of anti-lymphoma treatment regimen was mainly dependent on the pathological subtypes of NHLs. Tumor response was reviewed and classified according to the International Workshop Criteria.

**Results:**

A total of 78 patients were enrolled, among whom, 42 (53.8%) were with Diffuse large B cell Lymphoma (DLBCL), and 29 (37.2%) were with Burkitt lymphoma (BL). BL patients presented with higher risk features as compared with DLBCL in terms of numbers of extranodal diseases (*P* = 0.004) and poor Eastern cooperative oncology group (ECOG) score (*P* = 0.038). The estimated 2-year overall survival (OS) and progression free survival (PFS) rate was 74.3 ± 8.1%, 28.9 ± 11.0%, and 54.2 ± 8.1%, 19.2 ± 7.5% for DLBCL and BL, respectively. In multivariate analysis, international prognostic index (IPI) score was an independent prognostic factor for predicting both OS (OR = 2.172, 95% CI 1.579–2.987, *P* < 0.001) and PFS (OR = 1.838, 95% CI 1.406–2.402, *P* < 0.001).

**Conclusions:**

HIV associated NHLs represents a group of heterogeneous aggressive diseases with poor prognosis. IPI parameters were still effective in predicting the prognosis of HIV associated NHLs.

**Electronic supplementary material:**

The online version of this article (doi:10.1186/s13027-017-0120-2) contains supplementary material, which is available to authorized users.

## Background

Non-Hodgkin’s Lymphoma (NHL) is one of the most common types of malignancies with high morbidity and mortality in the patients who were infected by Human Immune-deficiency Virus (HIV) [1]. Generally, HIV associated NHLs present with more aggressive clinical behaviors as compared with general population, exemplified with a huge tumor burden, more extranodal diseases, and a tendency of involving genital system [2, 3]. Fortunately, although the prognosis of HIV associated NHLs remains very poor, with the introduction of combined antiretroviral therapy (cART) and high dose chemotherapy, a considerable portion of the patients could be cured [4, 5].

The International Prognostic Index (IPI) [6], which includes age, Eastern Cooperative Oncology Group (ECOG) performance status, Lactate Dehydrogenase (LDH) level, Ann Arbor stage, and extranodal involvement, is used extensively for evaluating patients with aggressive lymphomas, especially for those with diffuse large B cell lymphoma (DLBCL) and receive doxorubicin-containing chemotherapy such as R-CHOP [7, 8]. Although its role as prognostic factor in stratification of the NHL patients in daily practice and clinical trial is well established in general population, its validity remains controversial in HIV associated NHLs.

Another important issue is the complexity of the background of the HIV associated NHLs, such as more aggressive phenotype, immune-deficiency status. What even worse is that treatment factors (cART) also intervene the clinical outcome and prognosis [9–11]. Chronic antigen stimulation (HIV infection) could lead to a polyclonal B-cell expansion and finally promotes the emergence of monoclonal B cells. Both germinal center B cell-like (GCB) active B cell subtype (ABC) could be observed in HIV associated DLBCL. The frequency of double hit mutation could be observed in around 20% in HIV patients with NHLs [12–14]. Thus, a more comprehensive analysis incorporating above mentioned factors is warranted to give more precise stratification of HIV associated NHLs.

In China, with the increasing of incidence of HIV infection in recent years, acquired immunodeficiency syndrome (AIDS) associated NHL became more and more frequently. This subset of the patients might present distinct clinical behavior and treatment outcome as compared with their western counterpart. Hence, we performed this study to describe the clinical features and evaluate the potential prognostic factors of the Chinese AIDS patients with NHLs.

## Methods

### Patients

The HIV-infected patients who were newly diagnosed NHL were consecutively entered in this study from Jan.2002 to May.2015 in Shanghai Public Health Clinical Center. The subtypes of the lymphoma were defined according to the WHO 2008 classification system.

Systemic evaluation of the patients including complete blood cell count (CBC), biochemical parameters, LDH, viral panel (HIV, HBV, and HCV *etc.*), CD4 cell count, bone marrow and radiological examinations, was performed to stage the disease and assess the general and disease status.

### Anti-lymphoma treatment

Sixty-four out of 78 patients received anti-lymphoma treatment, and the regimen for the first line chemotherapy was mainly dependent on the pathological subtypes of lymphomas. For BL and PBL, Hyper-CVAD A (Cyclophosphamide [CTX] 300 mg/m^2^ Q12h × 6, D1-3, Dexamethasone [DEX] 40 mg/d, D1-4, D11-14, Vincristine [VCR] 1.4 mg/m^2^, D4, D11, Adriamycin [ADR] 50 mg/m^2^, D4) and B regimen (Methotrexate 1 g/m^2^, D1, Ara-C 2 g/m^2^ Q12h, D2-3) were given alternatively to the patients. For DLBCL, CHOP regimen (CTX 750 mg/m^2^, D1, ADR 50 mg/m^2^, D1, VCR 1.4 mg/m^2^, D1, Prednisone 60 mg/m^2^, D1) was initially designed for the patients, initiated from Dec.2014, to enhance the efficacy of young high risk patients (age < 60 years, IPI > 1). DA-EPOCH (Etopside 50 mg/m^2^ D1-4, VCR 0.4 mg/m^2^ D1-4, ADR 10 mg/m^2^ D1-4, all above continuous for 24 h, CTX 750 mg/m^2^ D5, DEX 40 mg D1-5) was also given to this subgroup of the patients [15]. The patients with FL and IBL received CHOP regimen the same as those with DLBCL. The detailed treatment regimen was depicted in Additional file 1: Table S1. No irradiation therapy was given concurrently during the treatment.

Rituximab 375 mg/m^2^ was designed to give to the patients with a suitable CD4 cell count (>50 cells/μl) to prevent the deep suppression of both B and T cell immunity. Since rituximab usage is not covered by medical insurance in most provinces in China, the application of treatment with rituximab also depended on patients’ willing. Fifty patients had a CD4 count greater than 50 (2 with PBL without CD20 expression), and 20 patients (40%) received rituximab.

The first line treatment regimen was discontinued if patients experienced any severe adverse event or lymphoma progressing. The initiation of the second line or salvage treatment regimen for relapsed and refractory patients was dependent on the decision of the physicians according to the condition of the patients.

### Anti-HIV treatment

ART was given immediately after the diagnosis of HIV with the exception of 5 patients due to rapid progression of lymphoma [16]. They were receiving either a combination of 2 nucleoside reverse transcriptase inhibitors with a protease inhibitor (*n* = 1) or with a non-nucleoside reverse transcriptase inhibitor (*n* = 70), or with an integrase inhibitor (*n* = 2).

### Response assessment

Evaluation of the efficacy was performed one month after completion of all the treatment. Thoracic, abdominal, and pelvic computed tomography scans were performed even if those areas were not initially affected [17, 18]. Interim staging was performed after completion of four cycles of treatment for DLBCL and FL patients. For BL and IBL patients, these evaluations were performed every 2 cycles. Tumor response was based on radiographic review and classified as complete response (CR), partial response (PR), overall response (OR, CR + PR), stable disease (SD), or progressive disease (PD) according to the International Workshop Criteria [19].

### Statistical analysis

Fisher’s Exact P test and one way ANOVA test were used to compare the clinical parameters in different subtypes of lymphoma. All patients entered into the study were followed up. Overall survival (OS) was measured from the date of disease diagnosis to death (failure) or alive at last follow-up (censored). Progression free survival (PFS) was defined as time from disease diagnosis to treatment failure such as relapse, progressive disease, any new anti-lymphoma treatment, and death of any reasons, or alive in CR at last follow-up (censored). Kaplan-Meier analysis was used to calculate the distribution of OS and PFS. Binary logistic regression was used for the multivariate analysis of prognostic parameters for response, while Cox model was used to identify prognostic variables for OS and PFS. A limited backward selection procedure was used to exclude redundant or unnecessary variants. To provide quantitative information on the relevance of results, 95% confidence intervals (95% CIs) of odds ratios (ORs) was calculated. All above statistical procedures were performed with the SPSS statistical software package, version 16.0.

## Results

### Patient characteristics

A total of 78 patients were enrolled, among whom, 42 (53.8%) were with DLBCL, 29 (37.2%) were with Burkitt lymphoma (BL), 5 (6.4%) were with plasmablastic lymphoma (PBL) and 1 (1.3%) was with follicular lymphoma (FL) and angioimmunoblastic lymphoma (IBL), respectively. The median age of the patients was 48 (24–74). A strong tendency of male patients was observed (89.7% vs. 10.3%). The clinical characteristics of the patients were shown in Table 1. The BL patients presented with higher risk features as compared with DLBCL patients in terms of numbers of extranodal disease (*P* = 0.004) and poor ECOG score (*P* = 0.038), while age (*P* = 0.151), gender (*P* = 0.692) and CD4 cell count (*P* = 0.526) were distributed equally in the two groups.

**Table 1:** Characteristics of the patients

Characteristics	DLBCL (*n* = 42)	BL (*n* = 29)	PBL and IBL (*n* = 6)
Age (years)			
Median	44	54	48
Range	24–73	25–73	33–74
	*P* = 0.151		
Gender (%)			
Male	37 (88.1)	27 (93.1)	5 (83.3)
Female	5 (11.9)	2 (6.9)	1 (6.7)
	*P* = 0.692		
Ann Arbor stage (%)			
I, II	23 (54.8)	10 (34.5)	3 (50.0)
III, IV	19 (45.2)	19 (65.5)	3 (50.0)
	*P* = 0.146		
Extranodal diseases (%)			
0,1	33 (78.6)	15 (51.7)	4 (66.7)
> 1	9 (21.4)	14 (48.3)	2 (33.3)
	*P* = 0.004		
LDH			
Median	334	373	283
Range	139–6016	178–5009	182–739
	*P* = 0.257		
ECOG score (%)			
0,1	18 (42.9)	5 (17.2)	3 (50.0)
> 1	24 (57.1)	24 (82.8)	3 (50.0)
	*P* = 0.038		
IPI (%)			
0,1	16 (38.1)	5 (17.2)	3 (50.0)
> 1	26 (61.9)	24 (82.8)	3 (50.0)
	*P* = 0.069		
CD4 cell count			
Median	106	108	31
Range	4–483	1–549	3–69
	*P* = 0.526		

33% (25/78) of the patients was confirmed to be infected with HIV before lymphoma diagnosis. 22% (17/78) of them received ART with a median treatment exposure of 12 months before the diagnosis of lymphoma, whereas 78% (61/78) of the patients were ART-naïve. 67% (53/78) of the patients were simultaneously diagnosed of lymphoma and HIV. Notably, 14% (11/78) of the patients had a plasma HIV-1 RNA viral load below 40 copies/ml at the time of lymphoma diagnosis. Table 2 summarized the baseline characteristics of these patients.

**Table 2:** Baseline characteristics of HIV condition of the patients

Characteristics	
AIDS before lymphoma diagnosis (n, %)	25 (33)
ART at lymphoma diagnosis (n, %)	17 (22)
ART exposure from lymphoma diagnosis (months)	12 (1–84)
Patients with CD4 count <200 cells/uL (n, %)	59 (75)
HIV RNA < 40 copies/ml at lymphoma diagnosis (n, %)	11 (14)

### Treatment response

Among 35 DLBCL patients, 22 (62.9%) and 26 patients (74.3%) achieved CR and OR, respectively. For 23 patients with BL, 5 (21.7%) achieved CR while 8 patients (34.8%) achieved OR. One patient with FL achieved PR. None of the patients with PBL and IBL obtained any response.

Further prognostic analysis for CR induction was performed in 58 patients with DLBCL or BL after anti-lymphoma treatment. As shown in Additional file 1: Table S2, in univariate analysis, DLBCL has significant higher CR rate than BL (*P* = 0.003). Ann Arbor stage (*P* < 0.001), number of extranodal disease (*P* = 0.001), ECOG performance status (*P* = 0.001), LDH (*P* = 0.008) and IPI (*P* < 0.001) were proved to be significantly associated the CR induction results.

IPI score and pathological types were entered into multivariate logistic regression analysis. It was proved that pathological types (DLBCL vs. BL, *P* = 0.038, OR = 0.240, 95% CI 0.063–0.925) and IPI score (*P* < 0.001, OR = 0.371, 95% CI 0.229–0.601) were independent prognostic factors of CR (Table 4).

### Survival analysis

**Fig. 1:**
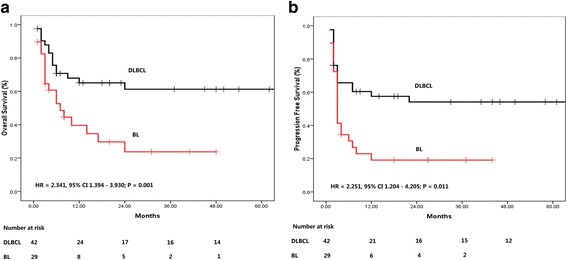
Kaplan-Meier curves for overall survival (OS) and progression free survival (PFS) of treated patients. **a** OS, (**b**) PFS

Among 64 patients who received anti-lymphoma treatment, the median follow up time was 13.5 months due to the poor prognosis of the patients. The estimated 2 year OS and PFS rate were 74.3 ± 8.1%, 28.9 ± 11.0%, 20.0 ± 17.9%, and 54.2 ± 8.1%, 19.2 ± 7.5%, and 0% for DLBCL, BL and PBL + IBL, respectively (Fig. 1). One FL patient who achieved PR relapsed after 1 year.

**Fig. 2:**
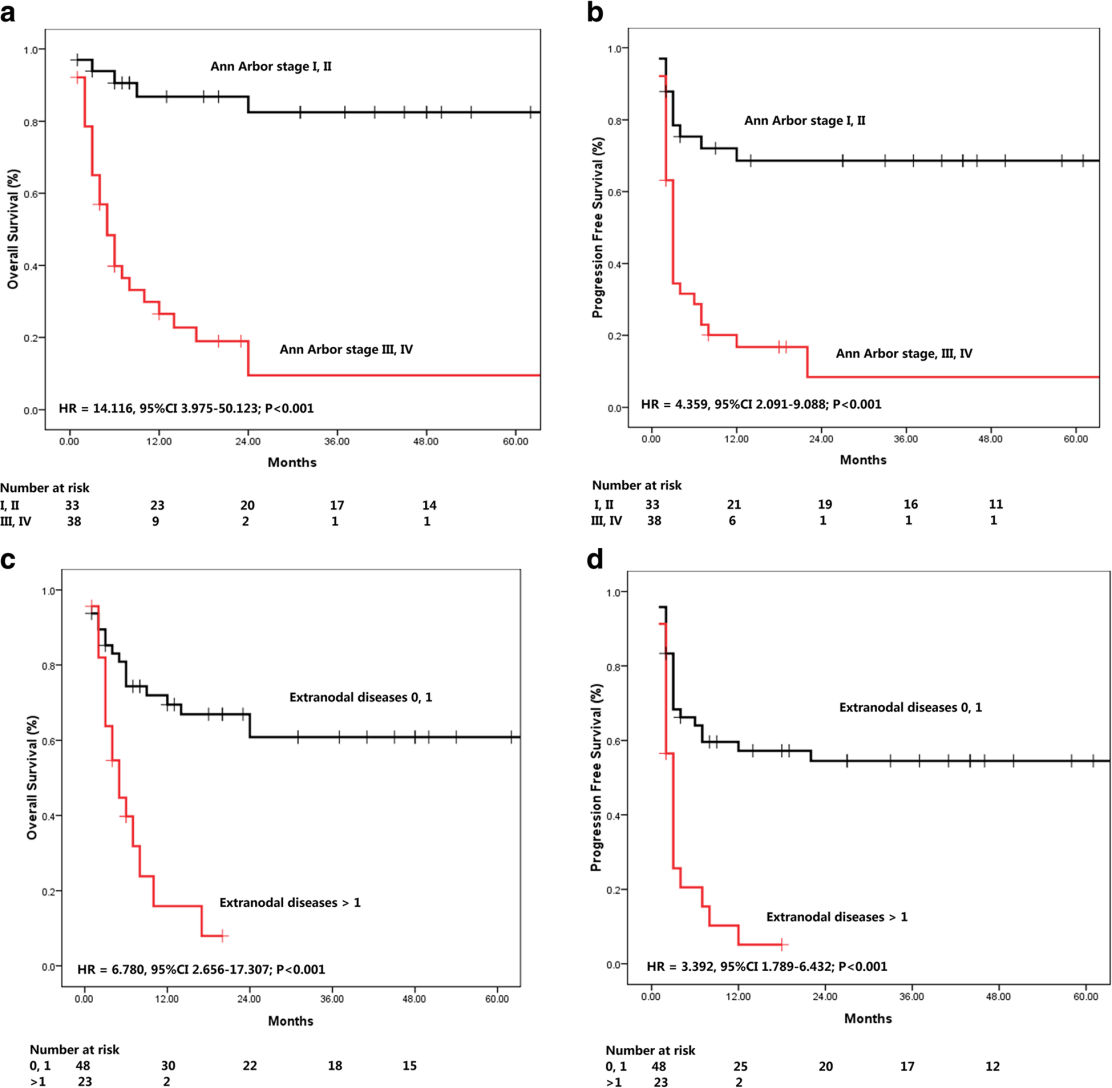
Univariate analysis of potential prognostic clinical factors. **a**, **b** Ann Arbor stage for OS and PFS. **c**, **d** Extranodal Diseases for OS and PFS

**Fig. 3:**
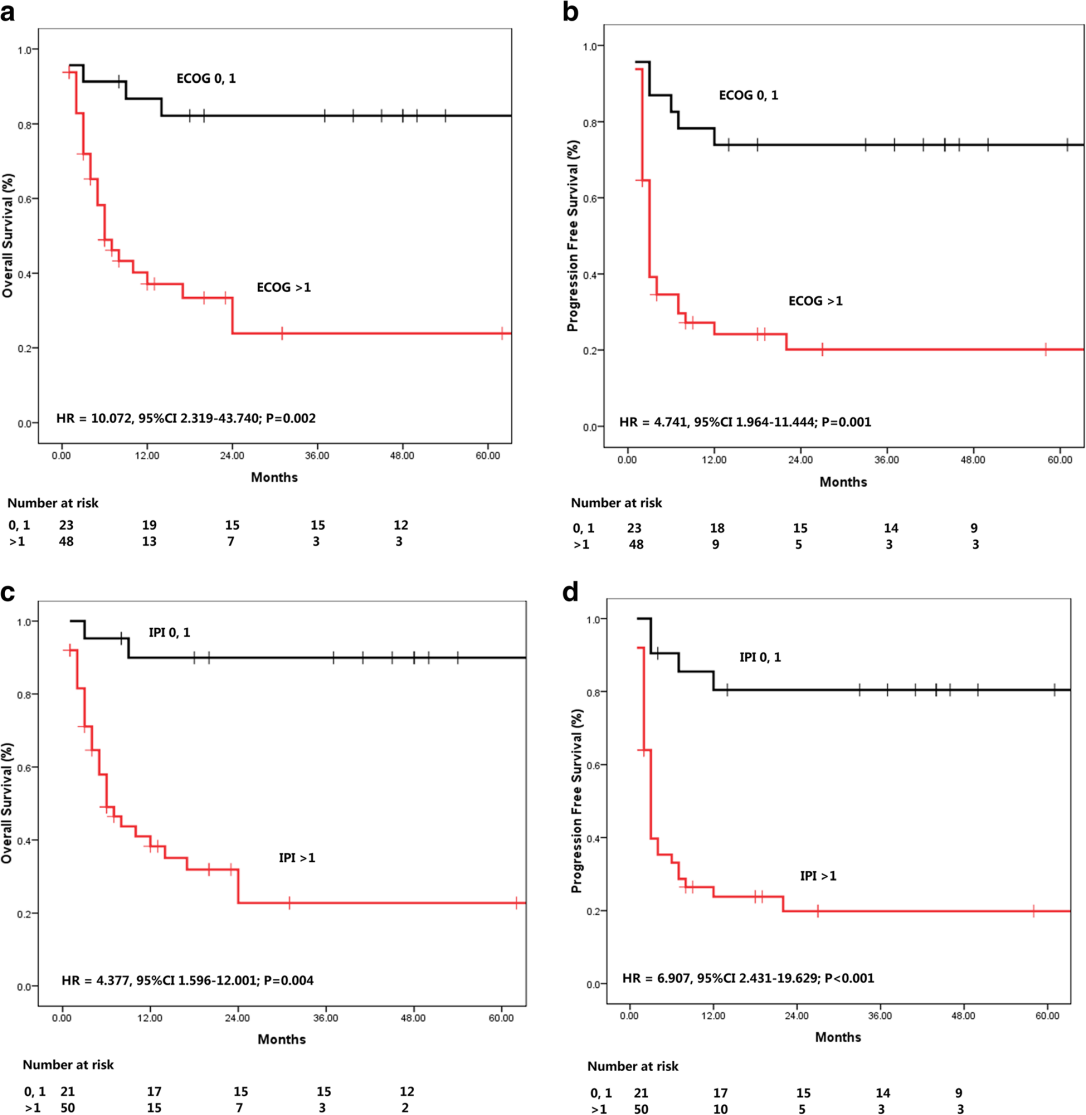
The usefulness of ECOG and IPI in predicting the prognosis of patients. **a**, **b** ECOG performance status for OS and PFS. **c**, **d** IPI for OS and PFS

Since a survival plateau could be observed in DLBCL and BL patients (Fig. 1), further prognostic analysis was performed in a group combining the two subsets of the patients. As shown in Figs. 2 and 3, Ann Arbor stage (I, II *vs.* III, IV), number of extranodal diseases (0, 1 *vs.* > 1), ECOG performance status (0,1 *vs.* > 1), and IPI score (0,1 *vs.* > 1) were all significantly associated the survival (OS and PFS) of the patients. LDH as continuous variable, could predict OS (HR = 1.000, 95% CI 1.000–1.001, *P* = 0.001) and PFS (HR = 1.000, 95% CI 1.000–1.001, *P* = 0.002), while CD4 cell count at diagnosis was not associated with the treatment outcome (OS: HR = 1.000, 95% CI 0.996–1.003, *P* = 0.824; PFS: HR = 0.998, 95% CI 0.992–1.005, *P* = 0.733). The estimated 2-year OS and PFS rates were shown in Table 3.

**Table 3:** The 2 year estimated OS and PFS rate in different subgroups

	OS (%)	PFS (%)
Ann Arbor stage		
I, II	88.6 ± 6.3	68.6 ± 8.3
III, IV	13.0 ± 10.3	8.4 ± 6.7
Extranodal diseases		
0, 1	71.7 ± 7.8	54.5 ± 7.4
> 1	10.3 ± 9.5	0
ECOG score		
0, 1	90.2 ± 6.6	73.9 ± 9.2
> 1	51.4 ± 9.0	20.1 ± 6.7
IPI score		
0, 1	95.0 ± 4.9	80.4 ± 8.8
> 1	49.6 ± 8.8	19.9 ± 6.4

In multivariate analysis, only IPI score was the independent prognostic factor for predicting both OS (OR = 2.172, 95% CI 1.579–2.987, *P* < 0.001) and PFS (OR = 1.838, 95% CI 1.406–2.402, *P* < 0.001) (Table 4).

**Table 4:** Multivariate analysis of potential prognostic factors for the patients

Variables	CR		OS		PFS	
	*P*	OR (95% CI)	*P*	OR (95% CI)	*P*	OR (95% CI)
IPI score	<0.001	0.371 (0.229–0.601)	<0.001	2.172 (1.579–2.987)	<0.001	1.838 (1.406–2.402)
Pathology subtype (DLBCL vs. BL)	0.038	0.240 (0.063–0.925)	NS	–	NS	–

## Discussion

HIV associated NHL represents a type of aggressive malignancies. Although intensive chemotherapy was given, the prognosis of the disease remained very poor. It was controversial whether some important prognostic factors in HIV negative counterpart such as IPI could still work in this special group of the patients [20]. In this study, we included a cohort of 78 patients to analyze the characteristics of AIDS associated NHL and to evaluate the prognostic factors of the disease.

Very similar to the normal HIV negative population, the most common subtype of lymphoma observed in HIV-infected patients was also DLBCL (42/78, 53.8%) [21]. As an important trigger of HIV to activate *c-myc*, BL was the second common pathologic subtype of the lymphoma (29/78, 37.2%) among this population. Compared to NHL in general population, AIDS patients with NHL presents with a more aggressive features including advanced Ann Arbor stage, significantly elevated LDH, and poor ECOG performance status, and a high IPI score eventually [2]. Of note, BL patients presented with even higher risk features while compared with those with DLBCL [22]. According to our data, BL patients manifested with the feature of more extranodal diseases (*P* = 0.004) but poorer ECOG scores (*P* = 0.038).

In this study, it was observed that the Chinese NHL patients presented with the characteristics of very low rate of ART exposure prior to the lymphoma diagnosis. Its negative impact on the anti-tumor treatment outcome could not be excluded. The severe immunosuppression status as well as the more aggressive disease phenotypes of these patients all strongly challenged the health care providers. One of the main reasons for the late ART initiation is that most of AIDS patients in China are from low income class, or so called “grass roots”, who are often less educated, single or separated from their family to work outside to strive. Although Chinese government have made great efforts to provide the basic education of AIDS, making the free ART publicly available, especially to this population, it seemed that a long way still needs to be run to popularize contemporary knowledge of HIV in such a large country with a huge population. The ignorance of the HIV infection of the patients and the prejudice from their family members should be firstly diminished. In this study, most of the patients came to the hospital not for suspicion of HIV infection, but for the complications, including but not limited to fever, unexplained symptoms and signs, or even hematological events like tumor or pancytopenia.

AIDS patients with DLBCL or BL could still achieve a survival plateau. However, it seemed that this plateau is significantly lower than that of the HIV negative counterpart, especially for BL patients [23]. In this study, the 2-year OS and PFS rates for DLBCL and BL were 74.3 ± 8.1%, 28.9 ± 11.0%, and 54.2 ± 8.1%, 19.2 ± 7.5%, respectively. High dose chemotherapy other than Hyper-CVAD A and B using the strategy including more anthracyclin (it was observed that most of BL patients progressed while received B regimen), might be designed. As for DLBCL, CHOP is not enough for the HIV-infected patients. An escalated regimen such as DA-EPOCH or CHOP-E should be considered for further clinical trial.

Various prognostic parameters have been used for predicting the treatment outcome of NHL [7, 11, 24]. Among them, the IPI is probably the most commonly used one [25]. Based on IPI, various systems such as FLIPI were designed for some special subtypes of lymphoma [24]. In this study, we have examined the efficacy of IPI parameters in HIV associated NHL (mostly BL and DLBCL). It was encouraging to prove that IPI parameters still worked in such kind of the patients, almost all the IPI parameters were useful in univariate analysis, and IPI was the independent prognostic factor for predicting both OS and PFS. Interestingly, the initial CD4 count before treatment was not associated with the treatment outcome of lymphoma in terms of CR rate, OS or PFS. It was traditionally believed that a low CD4 cell count was associated with death, which was proved in two large studies in pre-cART and early cART eras [26]. We believed that with the wide application of cART treatment, viral load and other related factors were abrogated.

## Conclusion

HIV associated NHLs represents a group of heterogeneous aggressive diseases with poor prognosis. Current anti-lymphoma treatment should be improved in this special group of patients in future clinical practice. IPI still works in the HIV-infected NHL patients in predicting the prognosis during the cART era. However, this study was a retrospective one and the treatment outcome of the patients, especially for BL, need to be further monitored. And the potential usefulness of existing clinical prognostic factors in AIDS patients should be further addressed in future studies.

## Additional file

Additional file 1: Table S1. Regimens of chemotherapy. **Table S2.** Univariate analysis of the factors affecting CR in DLBCL and BL patients. (DOCX 14 kb)

## Abbreviations

ABC: Activated B-cell subtype
ADR: Adriamycin
BL: Burkitt lymphoma
cART: Combined antiretroviral therapy
CBC: Complete blood cell count
CR: Complete response
CTX: Cyclophosphamide
DEX: Dexamethasone
DLBCL: Diffuse large B cell lymphoma
ECOG: Eastern cooperative oncology group
FL: Follicular lymphoma
HIV: Human immune-deficiency virus
IBL: angioimmunoblastic lymphoma
IPI: International prognostic index
LDH: LactateDehydrogenase
NHL: Non hodgkin’s lymphoma
OR: Overall response
ORs: Odds ratios
OS: Overall survival
PBL: Plasmablastic lymphoma
PD: Progressive disease
PR: Partial response
SD: Stable disease
VCR: Vincristine
