# Supplementary material for: Clinical and prognostic analysis of 78 patients with human immuno-deficiency virus associated non-Hodgkin’s lymphoma in Chinese population
Source: Infect Agent Cancer. 2017 Jan 23;12:7. doi: 10.1186/s13027-017-0120-2 (PMC5259913; doi:10.1186/s13027-017-0120-2)
Supplement: Additional file 1: Table S1. — Regimens of chemotherapy. Table S2. Univariate analysis of the factors affecting CR in DLBCL and BL patients. (DOCX 14 kb) [file 13027_2017_120_MOESM1_ESM.docx]

**Supplementary data**

**Supplementary Table 1. Regimens of chemotherapy**

| **Chemotherapy regimen** |  |
| --- | --- |
| **Hyper-CVAD A** | CTX 300mg/m^2^ Q12h x 6, D1-3  DEX 40mg/d, D1-4, D11-14  VCR 1.4mg/m^2^, D4, D11  ADR 50mg/m^2^, D4 |
| **Hyper-CVAD B** | MTX 1g/m^2^, D1  Ara-C 2g/m^2^ Q12h, D2-3 |
| **CHOP** | CTX 750mg/m^2^, D1  ADR 50mg/m^2^, D1  VCR 1.4mg/m^2^, D1  PRED 60 mg/m^2^, D1 |
| **DA-EPOCH** | Etopside 50mg/m^2^ D1-4  VCR 0.4mg/m^2^ D1-4  ADR 10 mg/m^2^ D1-4  (all above continuous for 24 hours)  CTX 750 mg/m^2^ D5  DEX 40mg D1-5 |

**Supplementary Table 2. Univariate analysis of the factors affecting CR in DLBCL and BL patients**

| **Potential factors** | **P value** |
| --- | --- |
| **Pathological type**  (DLBCL vs. BL) | 0.003 |
| **Age**  (<60 vs. >=60) | 1.000 |
| **Ann Arbor staging**  (1,2 vs. 3,4) | <0.001 |
| **Extranodal Diseases**  (0,1 vs. >1) | 0.001 |
| **ECOG**  (0,1 vs. >1) | 0.001 |
| **LDH** | 0.008 |
| **IPI**  (0,1 vs. >1) | <0.001 |
| **CD4 cell count** | 0.674 |
